# Fingolimod Enhances Oligodendrocyte Differentiation of Transplanted Human Induced Pluripotent Stem Cell-Derived Neural Progenitors

**Published:** 2018

**Authors:** Azadeh Yazdi, Akram Mokhtarzadeh Khanghahi, Hossein Baharvand, Mohammad Javan

**Affiliations:** a *Department of Physiology, Faculty of Medical Sciences, Tarbiat Modares University, Tehran, Iran. *; b *Department of Brain Sciences and Cognition, Cell Science Research Center, Royan Institute for Stem Cell Biology and Technology, ACECR, Tehran, Iran. *; c *Department of Developmental Biology, University of Science and Culture, Tehran, Iran.*

**Keywords:** Fingolimod (FTY720), Cell therapy, Neural progenitor cell, Cuprizone, Oligodendrocyte, Mouse

## Abstract

Multiple sclerosis (MS) is an autoimmune disease which affects myelin in the central nervous system (CNS) and leads to serious disability. Currently available treatments for MS mainly suppress the immune system. Regenerative medicine-based approaches attempt to increase myelin repair by targeting endogenous progenitors or transplanting stem cells or their derivatives. Fingolimod exerts anti-inflammatory effects and directly affects neural cells. In this study we assessed the effect of fingolimod on transplanted human induced pluripotent stem cell derived neural progenitors (hiPSC-NPs). hiPSC-NPs were labeled by green fluorescence protein (GFP) and transplanted into the corpus callosum of mice which were chronically demyelinated after cuprizone (CPZ) feedings for 10 weeks. The animals received fingolimod from 1 day prior to NPs transplantation via gavage as well as daily intraperitoneal cyclosporine A from 2 days before cell transplantation until the time of sampling. At either 7 or 21 days after NPs transplantation, the animals were sacrificed and their brains were histologically evaluated for the number of transplanted cells and their fate. In the animals treated with fingolimod, we observed higher numbers of NPs within the injection site compared to the animals who did not receive fingolimod showing that hiPSC- NPs were more efficiently differentiated to the oligodendrocyte lineage. These data have suggested that repetitive treatment with fingolimod, beside its anti-inflammatory effect, may enhance the survival and differentiation of transplanted NPs to oligodendrocyte lineage cells to participate in myelin repair.

## Introduction

Multiple sclerosis (MS) is a major inflammatory demyelinating disorder of the central nervous system (CNS) with oligodendrocyte degeneration and myelin loss which leads to disabilities (1). Demyelination plaques are detectable in different parts of the CNS of MS patients (2). Currently available treatments for MS predominantly consist of immune suppressants. In another approach, using regenerative medicine strategies, scientists have attempted to enhance myelin repair by efficient recruitment of endogenous neural progenitor cells or by transplantation of stem cells or their derivatives (3-5). Oligodendrocytes are responsible for developmental myelination and remyelination of CNS myelin insults (6). Remyelination requires generation of new mature oligodendrocytes that differentiate from oligodendrocyte progenitor cells (OPCs) and neural progenitors (NPs) (7). Several studies have reported the therapeutic advantages of stem cells in animal models of MS (3, 8). Pluripotent cells may form teratomas (9), therefore replacing strategies such as induction of OPCs from embryonic stem cells (ESCs) and the use of cells with induced pluripotency such as derived neural progenitors (iPSC- NPs) is a more attractive approach (10-13). Wang *et al.* and Yang *et al.* have separately reported that induced OPCs functionally restore myelin in animal models of hypomyelination (14, 15). Transplanted OPCs appeared to effectively repair the myelin sheets (16). In other research, NCs transplantation facilitated the process of remyelination (8, 17 and 18). In this study we transplanted NPs derived from iPSCs, as potential sources of OPCs and myelinating cells (18-20), into cuprizone (CPZ) induced demyelinated mice. 

Anti-inflammatory medications are the currently available disease modifying drugs that have been approved for treatment of relapsing-remitting MS (RRMS). Among these, fingolimod (FTY720) is the first oral medicine approved by the FDA in 2010 for treatment of RRMS. Fingolimod undergoes rapid phosphorylation *in-vivo* by sphingosine kinases (SPHK), especially SPHK2, to produce its active form (fingolimod phosphate). Fingolimod phosphate binds to sphingosin-1-phosphate receptors (S1P) on lymphocytes and prevents their exit from lymph nodes (21, 22). In addition to its anti- inflammatory effects, fingolimod exerts direct effects on different types of neuronal cells (23-26). Fingolimod has been shown to enhance OPCs differentiation to myelinating oligodendrocytes in an *ex-vivo* study (27) and accelerate myelin recovery after acute demyelination induced by CPZ (28). In experimental autoimmune encephalomyelitis (EAE), it promoted proliferation and differentiation of OPCs (29). Our research with a lysolecithin induced demyelination model showed that fingolimod increased OPCs recruitment and oligodendrogenesis (30). Fingolimod administration differentiated NPs to oligodendrocyte lineage cells (31) and increased survival (32), proliferation (33), differentiation, and migration (34) of OPCs *in-vitro*. In the current study, we reported the effect of systemic administration of fingolimod as a clinically available drug on the efficiency of transplanted human induced pluripotent stem cell derived neural progenitors (hiPSC-NPs) in a CPZ model of experimental demyelination. 

## Experimental

*Animals*

We purchased 7-week old male C57BL/6 mice from Pasteur Institute (Karaj, Iran). The animals were maintained in groups of 4 per cage with a 12-h light/12-h dark cycle. In total 50 mice were used in this study. All experiments were conducted in compliance with the NIH Guidelines for the Care and Use of Laboratory Animals. Tarbiat Modares University Committee of Ethics in Research approved the procedures. Efforts were made to minimize the animals’ suffering and reduce the numbers of animals used.

*Interventions*

After an acclimatization period of 7 days, the mice received 0.2% w/w CPZ (Sigma-Aldrich) in their standard chow for 10 weeks to induce chronic demyelination with very limited endogenous repair (35). CPZ administration was stopped after 10 weeks which equaled the time of cell transplantation. Ten mice per experimental group were used for the study. The Y-maze test was performed before and after CPZ administration and at day post-transplantation (dpt) 21. The animals received intraperitoneal injections of cyclosporine A (Novartis, S0056) from 2 days prior to cell transplantation until 7 or 21 dpt. 

**Figure 1:**
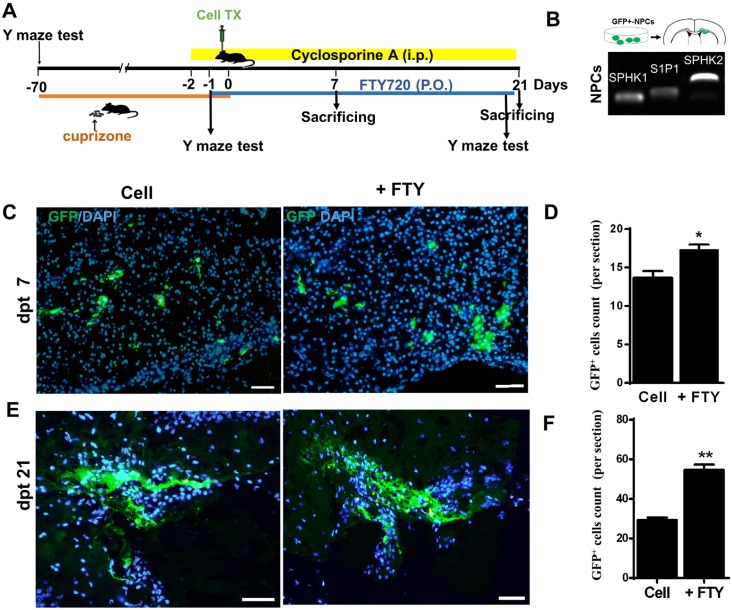
Human induced pluripotent stem cell derived neural progenitor (hiPSC-NP) transplantation and tracking. (A) Schematic diagram of the experimental procedure. (B) Sphingosine-1-phosphate receptor (S1P1) and sphingosine kinase (SPHK) gene expressions in NPs. (C-D) Tracking of transplanted cells in cell and cell +FTY treated groups and quantitative analysis of transplanted cell survival at 7 dpt. (E-F) Tracking of transplanted cells and quantitative analysis of transplanted cell survival at dpt 21. Cell (as control): animals that received NPs; +FTY: Animals that received fingolimod and NPs; ^*^P < 0.05 and ^**^P < 0.01; Scale bar: 50 µm. GFP: Green fluorescence protein (reporter gene); dpt: Day post-transplantation. n = 3

In order to evaluate the effect of fingolimod on transplanted cells, the animals received daily gavages of 0.3 mg/kg of fingolimod in water (Danesh Pharmaceutical Development Co., Tehran, Iran) in a volume of 100 µL. Fingolimod administration began one day prior to cell transplantation and continued until dpt 7 or 21. The 0.3 mg/kg/day oral dose was selected based on our previous study (30). Y-maze test were performed at dpt 21 before scarifying. In contrast to the experimental groups, the control animals received the same regime of CPZ, cyclosporine A, and cell transplantation, except fingolimod which was replaced by tab water gavages (Figure 1A).

*Y-maze test*

We conducted the Y-maze test to confirm the induction of demyelination in animals treated with CPZ and to study the effect on the treatments on behavioral changes in animals. This test is a simple behavioral test that measures spatial working memory and locomotor activity in animals by measuring spontaneous alternation and total entries to arms, respectively (36). The Y-maze test measured spontaneous alternations and entries to arms before initiation of CPZ feeding, at the end of 10 weeks of CPZ feeding prior to the fingolimod or tab water gavage and at dpt 21 before scarifying animals as previously described (36). Briefly, the Y-maze arms were designated as A, B, and C. Each mouse was placed randomly at the end of one of the arms and allowed to move freely for 8 min. The series of arm entries were recorded. The number of alternations was defined by the number of overlapping entrances (*e.g.*, BCA, CAB, ABC, BCA …). We calculated the percentage of alternations as the number of alternations to total number of entries -2 × 100 (37). Total arm entries were used as an index of ambulatory activity in the Y-maze, and the animals with scores below six entries were excluded. Only mice that showed reduced spontaneous alternations at day 0 were used for cell transplantation and the remainder of the procedures. Figure 1A show the experimental procedure.

*Cell transplantation*

After 10 weeks of CPZ feedings, the mice with demyelinated brains received cell transplants into their corpora callosa. The source of hiPSC-NPs used in this study was hiPSC line 8 (Royan hiPSC8, passage 22) (38) which were differentiated to neural progenitors, as characterized and GFP labeled by Starian *et al.* (39). Green fluorescence protein (GFP) positive hiPSC-NPs were previously characterized and transplanted into the demyelinated optic chiasm for remyelination (39). Labeled hiPSC-NPs (10^5 ^cells/2 µL of DMEM/F12 were injected into each corpus callosum at AP: -1.06, L: 0.6, and DV: 1.5 from bregma (Figure 1A) (40). The animals were sacrificed at dpt 7 or 21 to determine the number and fate of the transplanted cells.

*Tissue processing and staining*

At different time points after transplantation, we re-anesthetized the animals with chloral hydrate (480 mg/kg) after which they underwent transcardial perfusions with PBS followed by 4% paraformaldehyde. The animals’ brains were removed, post-fixed overnight in 4% paraformaldehyde, and sectioned into 6-μm coronal sections by a cryostat device (Histo-Line Laboratories, Milan, Italy). We obtained sections from -0.7 mm (bregma) to -1.34 mm (bregma) (41) for the following studies. 

To determine the fate of the cells after transplantation, immunostaining was performed as previously described (42). Briefly, the sections were incubated in Triton X-100, blocked with normal goat serum, and incubated overnight in primary antibody at 4 °C. Staining against GFAP, Olig2 and NeuN were used to identify astrocytes, OPCs, and neurons, respectively. Antibody against PLP was used to demonstrate myelination and anti-GFP antibody to identify the transplanted cells. After washing with PBS, the sections were incubated with secondary fluorescence-labeled antibody for 1 h at room temperature. Table 1 shows the characteristics of all applied primary and secondary antibodies. The sections were finally mounted with UltraCruz™ mounting medium that contained DAPI (Santa Cruz Biotechnology Inc., CA, sc-24941). We used ImageJ software to quantify the numbers of immunostained cells or extent of demyelination. A total of 3 sections per animal were stained, analyzed, and averaged. Data were entered into a final average for 3 animals per experimental group. 

For FluoroMyelin™ staining, we incubated the rehydrated cryosections from the corpora callosa in staining solution for 20 min according to the manufacturer’s protocol (Molecular Probes, F34652). The images were obtained with a BX51 Olympus microscope and captured by an Olympus DP72 camera for analysis.

**Table 1:** List of primary and secondary antibodies

**Antibody**	**Host**	**Cat. no.**	**Dilution**	**Label**
PLP	Rabbit polyclonal IgG	Abcam, Inc. (ab28486)	1:200	-
Olig2	Rabbit polyclonal IgG	Abcam, Inc. (ab81093)	1:200	-
GFAP	Rabbit polyclonal IgG	Dako(Z0334)	1:300	
GFP	Mouse monoclonal IgG	Abcam, Inc.(ab1218)	1:400	
NeuN	Rabbit monoclonal IgG	Abcam, Inc. (ab177487)	1:500	-
Anti-rabbit IgG	Goat anti-rabbit IgG	Life Technology(A-11011)	1:1000	Alexa Fluor 568
Anti-mouse IgG	Goat anti-mouse IgG	Life Technology(A-11001)	1:1000	Alexa Fluor 488

*RNA extraction and semi-quantitative PCR*

Total cellular RNA was extracted by the TRIzol reagent (Sigma-Aldrich) from NP cells. A total of 2 µg of purified RNA was run on the agarose gel to confirm the integrity of isolated RNA. cDNA was synthetized using RT PreMix (Bioneer) as a reverse transcription reagent and Oligo-dT primer. Table 2 shows the specific primers to the human sequences for semi-quantitative PCR and electrophoresis.

**Table 2:** List of primers used in RT-qPCR studies

**mRNA**	**Product length**	**Sequences**
S1P1	114	5'- TTCTGCGGGAAGGAAGTATG -3'
		5'- TGCTGCCGTTGTGTAGTTTC -3'
SPHK1	103	5´- GAGAAGTATCGGCGTCTGGG -3´
		5´- CTACAGGGAGGTAGGCCAGT -3´
SPHK2	200	5´- AGACGTGATGCTGGAAGGTG -3´
		5´- AGGGCGACGCGTAAAATAGA -3´

*FluoroMyelin™ staining*

We incubated the rehydrated cryosections from the animals’ brains prepared at the level of the cell injection site in staining solution for 20 min according to the manufacturer’s protocol (Molecular Probes, F34652). The sections were prepared from control, CPZ, CPZ+cell transplantation, CPZ+fingolimod, and CPZ+cell+fingolimod treated animals. The images were captured using an Olympus DP72 camera for analysis. The percentage of demyelination was assessed by using ImageJ software as the percentage of demyelination to the total area of the corpus callosum. Three sections per animal and three animals per experimental group were evaluated.

*Statistical analysis*

For cell counts unpaired *t*-test was used. Spontaneous alternations and total entries to the arms were analyzed using paired *t*-tests. *P*-values less than 0.05 were considered statistically significant. 

## Results

*Sphingosine-1-phosphate receptor (S1P1) and sphingosine kinase (SPHK) expression in human induced pluripotent stem cell derived neural progenitors (hiPSC-NPs)*

*In-vivo* phosphorylation of fingolimod by SPHK results in fingolimod phosphate, the active form of fingolimod (43). Fingolimod phosphate initially activates the S1P1 receptor via high-affinity receptor binding (44, 45). We have sought to evaluate the possible response of transplanted hiPSC-NPs to fingolimod and assess the capability of these cells for fingolimod phosphorylation. RT-PCR analyses of the expressions of SPHK and S1P1 receptor showed that hiPSC-NPs expressed SPHK1 and SPHK2, as well as the S1P1 receptor (Figure 1B). 

*Tracking the survival of transplanted cells*

We transplanted GFP positive hiPSC-NPs into demyelinated corpora callosa to evaluate their survival with or without fingolimod. At 7 days after transplantation, we observed detectable levels of GFP positive NPs at the injection site in the fingolimod treated and control groups (Figure 1C). The cells were also detectable at dpt 21 (Figure 1E). A comparison of the groups showed higher numbers of GFP positive cells in the fingolimod treated group at both dpt 7 (*P *< 0.05) and 21 (*P *< 0.01; Figures 1D and 1F). 

*The fate of engrafted neural progenitor (NP) cells*

**Figure 2:**
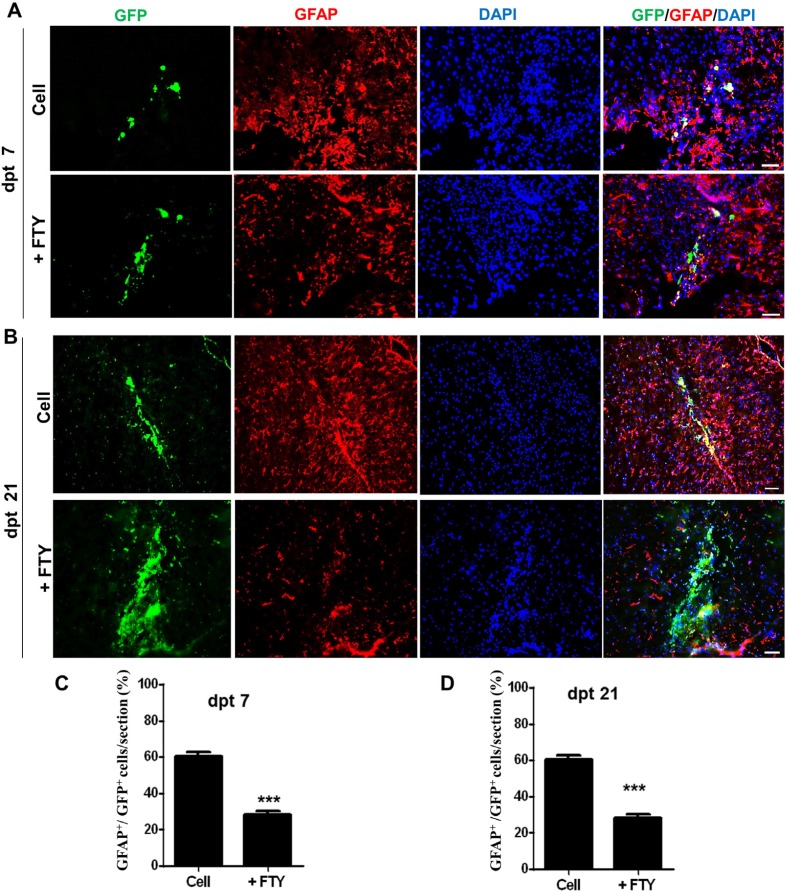
Evaluation of the effect of fingolimod on transplanted neural progenitor (NP) differentiation to astrocytes. (A) Transplanted NPs that expressed GFAP as an astrocyte marker at dpt 7. (B) Transplanted NPs that expressed GFAP at dpt 21. (C) Quantified data for transplanted NPs that expressed GFAP at dpt 7. (D) Quantified data for transplanted NPs that expressed GFAP at dpt 21. Control: Intact animals; CPZ: Animals that received cuprizone for 10 weeks. Cell (as control): animals that received NPs; +FTY: Animals that received fingolimod and NPs. ^**^P < 0.01 and ^***^P < 0.001. Scale bar: 50 µm. GFP: Green fluorescence protein (reporter gene); DAPI: Nuclei stain; GFAP: Astrocyte marker; dpt: Day post-transplantation. n = 3

We evaluated the effect of fingolimod on the fate of GFP positive transplanted NPs. Immunofluorescence staining against different markers specific to various types of neural cells was performed at days 7 and 21 after transplantation. Co-immunofluorescence staining against GFP and GFAP antigens showed that at dpt 7, 60.67 ± 2.33% of GFP positive cells in the control and 28.33 ± 1.76% of GFP positive cells in the fingolimod (*P* < 0.001) groups differentiated to the astrocyte-like cells (Figures 2A and 2C). At dpt 21, 51.67 ± 2.33% of GFP positive cells in the control and 31 ± 3.46% of GFP positive cells in the fingolimod treated groups (*P* < 0.001) differentiated to the astrocyte-like cells (Figures 2B and 2D). 

**Figure 3:**
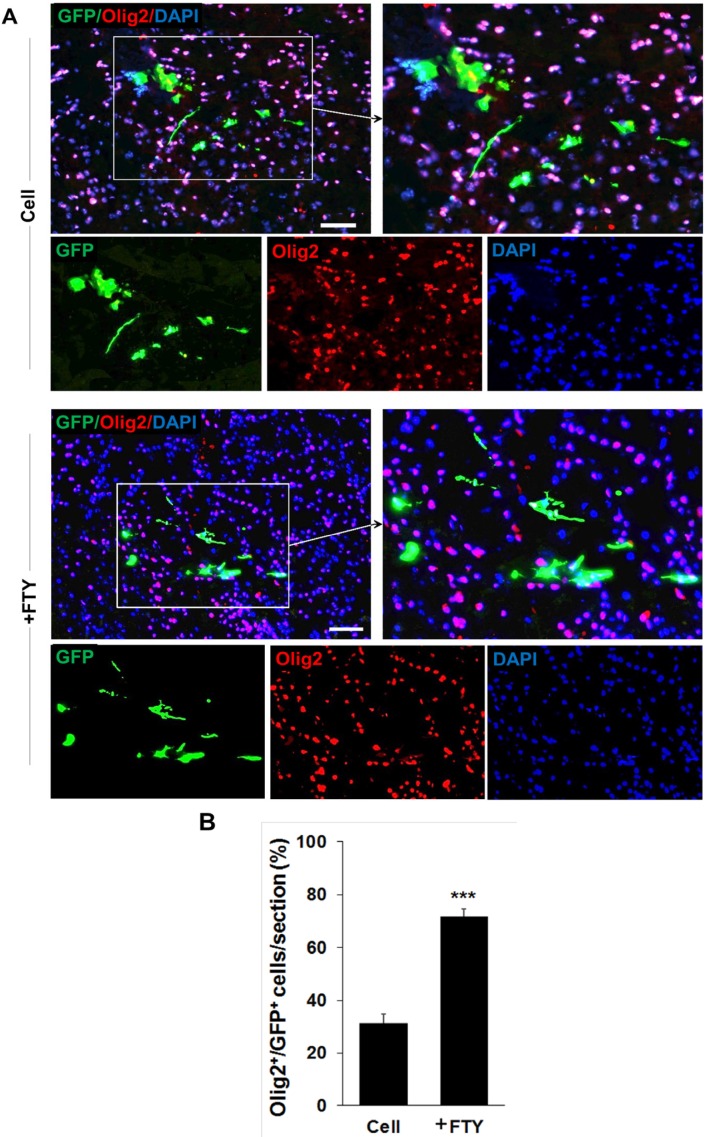
Evaluation of the effect of fingolimod on transplanted neural progenitor (NP) differentiation to oligodendrocyte lineage cells. (A) Transplanted NPs that expressed Olig2 as an oligodendrocyte lineage marker on dpt 7. (B) Quantitative analysis of transplanted cells that differentiated to oligodendrocyte lineage cells at dpt 7. Cell (as control): animals that received NPs. +FTY: Animals that received fingolimod and NPs. ^***^P < 0.001. Scale bar: 20 µm. GFP: Green fluorescence protein (reporter gene); DAPI: Nuclei stain; Olig2: Oligodendrocyte lineage marker; dpt: Day post-transplantation. n = 3

**Figure 4:**
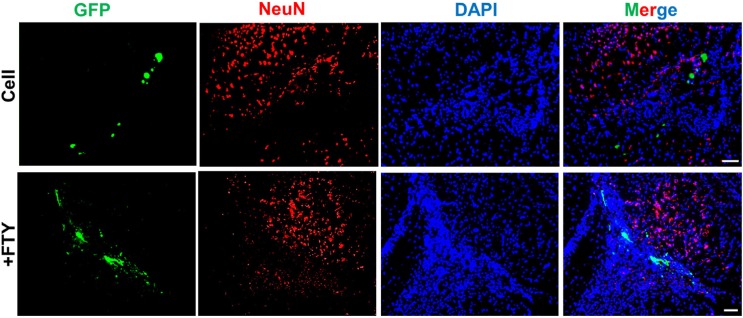
Evaluation of the effect of fingolimod on transplanted neural progenitor (NP) differentiation to neurons. No remarkable NeuN+/GFP+ cells were observed at dpt 7. +FTY: Animals that received fingolimod and NPs. Scale bar: 50 µm. GFP: Green fluorescence protein (reporter gene); DAPI: Nuclei stain; NeuN: Neuronal marker; dpt: Day post-transplantation. n = 3

Double staining with anti-GFP and anti-Olig2, as an oligodendrocyte lineage marker, on dpt 7 samples showed a significantly higher tendency for differentiation of transplanted NP cells to differentiate to oligodendrocyte lineage cells in the fingolimod treated group (67.5 ± 2.9) compared to the control group (32.1 ± 3.5; *P* < 0.001, Figures 3A and 3B). Immunostaining with anti-NeuN antibody at dpt 7 showed that none of the transplanted cells differentiated to neurons (Figure 4). 

*Effect of fingolimod on myelination by engrafted neural progenitor (NP) cells*

**Figure 5:**
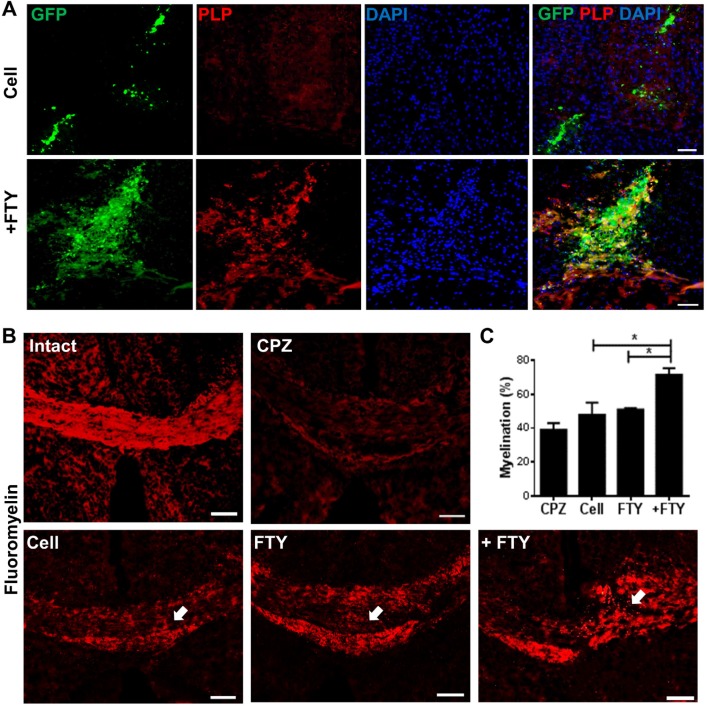
Evaluation of the effect of fingolimod on remyelination efficacy of transplanted neural progenitors (NPs). (A) Transplanted NPs expressed PLP as a mature oligodendrocyte marker at dpt 21. (B) FluoroMyelin™ staining showed the extent of myelination at dpt 21 at the injection site (arrows). (C) Quantification analysis of the extent of myelination at the cell injection site. ^*^P < 0.05; +FTY: Animals that received fingolimod and NPs; CPZ (as control): Animals that received cuprizone for 10 weeks; FTY: Animals that received fingolimod without cell transplantation; Scale bar in (A): 50 µm and (B): 100 µm. GFP: Green fluorescence protein (reporter gene); DAPI: Nuclei stain; PLP: Mature oligodendrocyte marker; dpt: Day post-transplantation. Arrows represent the cell injection site. n = 3

We compared the potency of full maturation of engrafted cells to myelinating oligodendrocytes in fingolimod treated and non-treated animals by labeling against GFP and PLP (as a mature oligodendrocyte marker) on dpt 21. The evaluation of each corpus callosum area within brain sections prepared from the injection site showed an apparent increase in PLP+/GFP+ cells in animals that received fingolimod (Figure 5A). The extent of myelination was evaluated by FluoroMyelin™ staining. Sections obtained from animals treated with fingolimod showed increased myelination at the engraftment site (Figure 5B). Quantification of the extent of demyelination (Figure 5C) indicated that animals which received both cells and fingolimod had higher levels of myelination at the injection site compared with sections prepared from animals that received cells or fingolimod alone (*P *< 0.05).

*Effect of fingolimod on behavioral changes following NPs engraftment*

**Figure 6:**
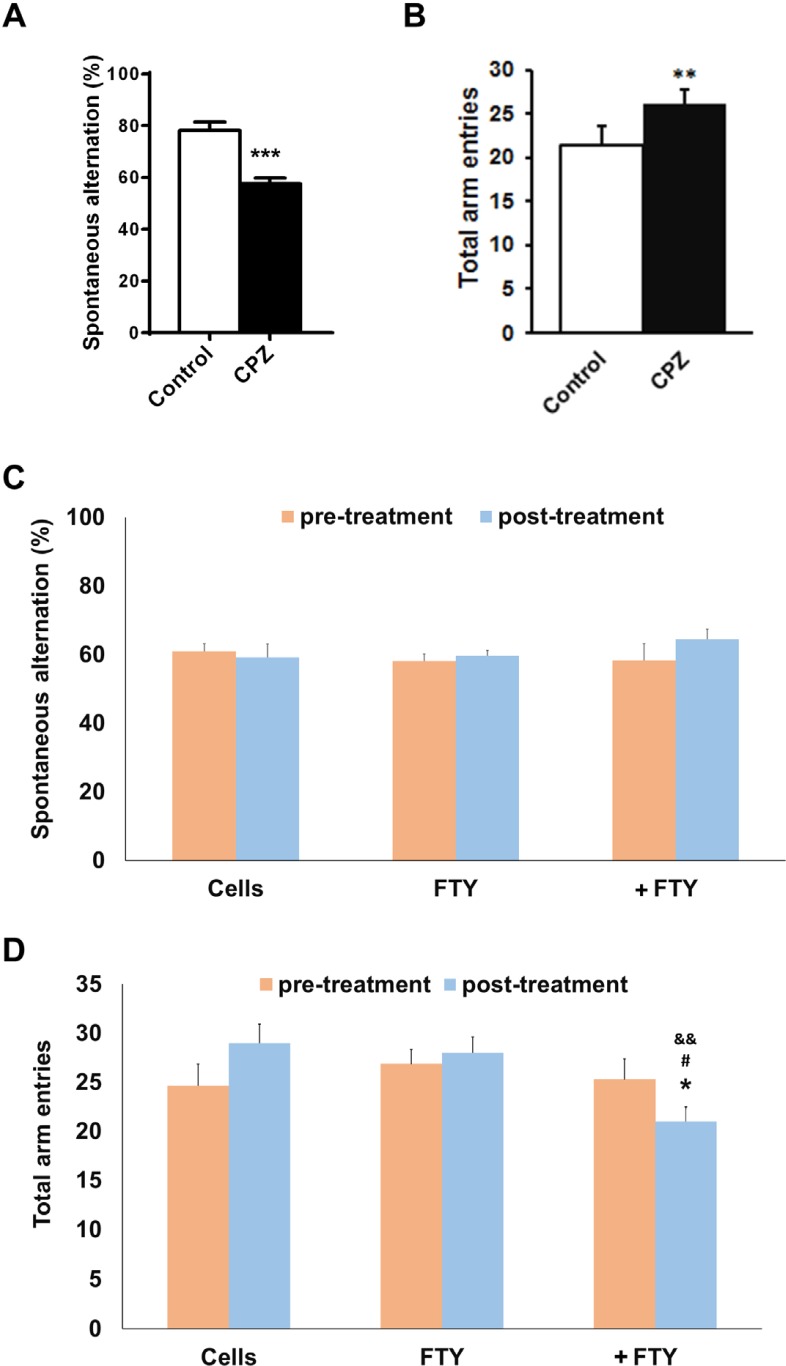
Evaluating the effect of fingolimod on Y-maze behavioral parameters in cuprizone-induced demyelinated mice transplanted with neural progenitors. Animals received cuprizone (CPZ) for 10 weeks, then transplanted with human induced pluripotent stem cell derived neural progenitors (hiPSC-NPs). Y-maze test was performed prior to CPZ, 10 weeks after CPZ feeding and 21 days after NPs transplantation with or without fingolimod (FTY) treatment. (A) Changes in spontaneous alternations following 10 weeks of CPZ feeding. (B) Changes in total arm entries following 10 weeks of CPZ feeding. (C) Changes in spontaneous alternations in CPZ-treated animals following 21 days of treatments. (D) Changes in total arm entries in CPZ-treated animals following 21 days of treatments. Cell: animals that received NPs; FTY: Animals that received fingolimod without cell transplantation; +FTY: Animals that received fingolimod and NPs transplantation; ^*^P < 0.05, ^**^P < 0.01, ^***^P < 0.001, compared to pre-treatment, ^&&^*P* < 0.01 compared to Cells, ^#^*P* < 0.05 compared to +FTY

Mice with induced demyelination by ten weeks cuprizone deeding, had impaired spatial working memory as measured by spontaneous alternations in Y-maze test (*P* < 0.01). Fingolimod did not possess a significant effect of the behavioral outcomes. The improved spontaneous alternations by fingolimod+NPs was not significant but locomotor activity as measured by the number of total arm entries was significantly restored toward control level (Figure 6).

## Discussion

Currently, common treatments for MS suppress the immune system in an attempt to decrease myelin damage (46). Fingolimod bind to different subtype of S1P receptors (47) and activates the lymphocyte S1P1 receptors, and prevents lymphocytes from exiting the lymph nodes and reduces their entry into the CNS (48). Fingolimod crosses the blood–brain barrier and exerts a direct effect on CNS cells (23). Some previous studies showed that fingolimod target S1P1 receptor on astrocytes and neural progenitors and/or binds with S1P5 on oligodendrocytes, oligodendrocyte progenitors and neurons (25, 27, 49 and 50). Treatments that benefit not only the immune system to reduce inflammation but also the CNS to promote neuroprotection and repair are desirable for MS disease (46, 51). NPs show promising potential for remyelination and exert neurotrophic properties (52-55). Any cell therapy approach that repairs myelin seems to be applicable in conjunction with immunosuppressive drugs that prevent myelin damage. Therefore, it is of immense interest to understand how immunosuppressive drugs interact with transplanted cells. Previous reports have shown that fingolimod could exert a beneficial effect on endogenous cell-mediated remyelination (30, 56 and 57). In this study we assessed the effect of fingolimod on hiPSC-NPs transplanted to the demyelinated corpora callosa of mice to observe its impact on survival and differentiation of transplanted hiPSC-NPs. Previously, we reported the potential of these transplanted cells to differentiate to myelinating cells (18). We showed that hiPSC-NPs expressed the S1P1 (receptor with the highest affinity to fingolimod) mRNA and potentially this receptor mediates its effects on NPs while we cannot rule out the role of other receptor subtypes. Previous reports showed NPs expression of S1P1 receptors which contributed to their migration toward the injury site (25). 

CPZ induced demyelination in the current study is a useful model to evaluate the remyelination process without interference by inflammatory reactions (58). Weak endogenous remyelination after 10-12 weeks of CPZ administration makes the model appropriate to study the differentiation and remyelination potency of transplanted cells in the presence or absence of fingolimod. Previous studies showed CPZ-fed mice displayed abnormal behavior in the Y-maze test during the demyelination and there was recovery of behavioral function during the remyelination process (59). Y-Maze as a behavioral test was used for evaluating locomotor activity (the number of arm entries) and spatial working memory (spontaneous alternation) (36, 60). Compared to normal, the cuprizone-treated mice showed increased locomotor activity and decreased alternations (59). These behavioral changes, helped us to select animals with demyelination in their corpora callosa for cell transplantation. 

Our data showed that at both dpt 7 and/ or 21 fingolimod-treatment, the animals had higher numbers of GFP labeled cells. Such differences between dpt 7 and 21 can be due to the proliferation of NPs. Piltti *et al.* at 2015 showed that engrafted human central nervous system derived neural stem cells (hCNS-SCns) proliferated at the side of transplantation (61). Meanwhile the increased proliferation was seen in iPSC-NPs transplantation in ischemic stroke animal model (62, 63). Another explanation for the higher numbers of the cells in fingolimod versus control group could be the consequence of the protection and reduced cell death. Coelho *et al.* found that fingolimod had a direct protective effect on OPCs via activation of Akt and extracellular signal-regulated kinase1/2 (64). Fingolimod also protected the affected axons from degeneration after induction of CPZ mediated demyelination (65). In a previous report, S1P increased proliferation and induced morphological changes in embryonic NPs (33). Under culture conditions, fingolimod promoted proliferation of the neural stem cells (66). Therefore, both protection and enhanced proliferation might contribute to the effect of fingolimod on the numbers of transplanted hiPSC-NPs that remained at dpt 7 and 21.

NPs can differentiate to various neural cell types, including oligodendrocyte lineage cells, depending on the surrounding niche. We chose the 0.3 mg/kg dose of fingolimod according to our previous study results in order to evaluate its effect on hiPSC-NPs differentiation. 

At both dpt 7 and 21, we have observed that significant numbers of the transplanted cells in the control group differentiated to astrocytes. Astrocytes are known as the main component of the glial scars which occur in brain injuries and neurodegenerative diseases such as MS (25). Thus, application of NPs in demyelinating insults may worsen the scares by differentiation of the transplanted cells to astrocytes. Concomitant treatment with fingolimod significantly reduced the number of astrocytes differentiated from NPs; therefore, fingolimod appeared to be a promising treatment for prevention of astrocyte differentiation from NPs. 

Our results showed a significant increase in Olig2 positive cells in fingolimod treated animals at dpt 7 and 21. These data implied that fingolimod significantly enhanced differentiation of transplanted NPs to oligodendrocyte lineage cells. A previous *in-vitro* study by Bieberich *et al.* showed that NPs exposed to fingolimod or other S1P1 analogues differentiated to OPCs. Fingolimod increased both the bioavailability and differentiation potency of OPCs (31, 32). This report supported the current study results of the effect of fingolimod on differentiation of transplanted NPs to the oligodendrocyte lineage cells. Both PLP immunostaining and FluoroMyelin™ staining at dpt 21 showed that GFP positive engrafted NP cells differentiated to myelinating oligodendrocytes within the corpus callosum. This confirmed that fingolimod at the applied dose enhanced the maturation of oligodendrocytes and myelin formation. CPZ-induced chronic demyelination produced remarkable hyperactivity and spatial working memory deficits. During the recovery, the number of arm entries was decreased by fingolimod+NPs transplantation treatment. These results showed beneficial effect of the treatment on behavior of mice. Increased locomotor activity seen in cuprizone-fed mice may be a consequence of mild myelin damage in some neural pathways (60). This finding may support for the improved behaviors following differentiation if NPs to oligodendrocytes. A21 dpt, transplanted cells did not express NeuN neuronal marker as a marker for mature neurons. No remarkable NeuN+/GFP+ cells at dpt 21 rule out the differentiation of NPs toward neuronal fate. The results showed that co-administration of fingolimod with NP cells therapy did not destroy NP survival and differentiation. Rather, it enhanced NP survival and reduced the risk of glial scar extension by decreasing the possibility of NPs differentiation to astrocytes and enhanced NP differentiation to myelinating cells in chronic CPZ induced demyelination.

These data have suggested that repetitive treatment with fingolimod, beside its anti-inflammatory effect, may enhance the survival and differentiation of transplanted NPs to oligodendrocyte lineage cells to participate in myelin repair. Fingolimod increased the NPs transplantation efficiency for remyelination. By in-depth studies on cell transplantation benefits in patients with multiple sclerosis, NPs transplantation with repetitive fingolimod administration seems a promising approach for controlling patients’ symptoms. 
